# Numerical, Theoretical, and Experimental Analysis of LVL-CFRP Sandwich Structure

**DOI:** 10.3390/ma17010061

**Published:** 2023-12-22

**Authors:** Michał Marcin Bakalarz, Paweł Grzegorz Kossakowski

**Affiliations:** Department of Theory of Structures and Building Information Modeling (BIM), Faculty of Civil Engineering and Architecture, Kielce University of Technology, Al. Tysiaclecia Panstwa Polskiego 7, 25-314 Kielce, Poland; mbakalarz@tu.kielce.pl

**Keywords:** carbon fiber, composites, core, laminated veneer lumber, reinforcement, sandwich structure, wood structures

## Abstract

Optimization of structural elements via composition of different components is a significant scientific and practical point-of-view problem aimed at obtaining more economical and environmentally friendly solutions. This paper presents the results of a static work analysis of small-size laminated veneer lumber (LVL) beams reinforced by a Carbon Fiber Reinforced Polymer (CFRP) sheet. The nominal dimensions of LVL beams were 45 × 45 × 850 mm, and 0.333- and 0.666-mm thick reinforcement layers were used. The reinforcement was applied on opposite sides of the cross section obtaining a sandwich-type structure. An epoxy resin was used as a bonding layer. The bending tests were conducted in the so-called four-point bending static scheme in edgewise and flatwise conditions. The results of experimental tests confirmed the validity of this combination of materials. The highest load-bearing capacity was obtained for configuration, where CFRP sheets with a thickness of 0.666 mm were placed on the sides of the core, parallel to the direction of loading and the veneer’s grain in the core. The increase in this case was up to a maximum of 57% compared to the core alone. The highest bending stiffness increase, 182% compared to the core alone, involves placing two layers of sheets perpendicular to the direction of loading, i.e., on the upper and lower surfaces. The presented novel sandwich structure can be competitive against traditional steel and reinforced concrete elements in civil engineering and can be utilized as beams or slabs.

## 1. Introduction

The pursuit of optimizing the structural elements of building objects by combining materials with different properties is the subject of numerous scientific papers. This combination results in a composite with improved or different properties compared to the materials used to create it. One such type of composite is a sandwich structure built with a lighter core, a bonding layer, and a surface material. Different materials can be applied as a core such as foams (polymer [1], metallic [2]), honeycomb [3], tubular [4], or wood.

For wood-based cores, energy absorption and fire performance were discussed by researchers. Energy-absorption characteristics of a sandwich structure made of coconut mesocarp and FRP skin (using glass and carbon fiber sheets) were presented by Liu et al. [5]. They obtained higher specific energy absorption for glass skin in comparison to metal or aluminum skin. Anjang et al. [6] described a model for calculating the mechanical properties of fire-exposed sandwich structures based on an analysis of E-glass/vinyl ester laminate and a balsa wood core with post-fire properties. They revealed that the thermal decomposition of the fire-exposed external layer is the key factor for decreasing the mechanical properties of the sandwich structure. Goodrich et al. [7] investigated thermal softening response and thermal recovery behavior for balsa wood cores. The temperature range to avoid permanent heat damage for the balsa core was revealed in the study.

Other parameters studied regarding the wood-based sandwich structures were mechanical properties. Monti et al. [8] discussed some of the mechanical properties, including quasi-static and fatigue tests, of flax fiber-balsa sandwich structures with the aim of possible industrial applications. Basha et al. [9] compared the behavior of balsa and birch wood cores and CFRP skin in terms of impact and post-impact response.

A detailed review of sandwich structure with a wood-based core, on the basis of balsa wood core, is presented by Galos et al. [10]. Overall, the state-of-the-art of sandwich structures is presented in [11].

In civil engineering, a common way to rehabilitate, strengthen, and optimize a new and existing structure is by adding an additional element. These additional elements can be made of steel [12], aluminum, or composite materials such as composites reinforced with aramid, glass [13], basalt, and carbon fibers [14,15]. The reinforcement can be positioned symmetrically or asymmetrically in the tensile or/and in the compression zone. The reinforcement can be placed inside and outside the cross-section and connected with wood by means of mechanical and adhesive connections.

Sokolovic et al. [16] analyzed the possibility of improving the mechanical properties of layered glued veneer modified with carbon fiber-reinforced fabrics during production, achieving a significant increase in the system’s load-bearing capacity and stiffness. Similar considerations were conducted by Percin and Uzun [17], who investigated various configurations of placing carbon fabric on the rupture modulus and elasticity modulus of small veneer samples made of thermally treated beech. Núñez-Decap et al. [18] verified the possibility of applying sheets made of basalt and carbon fibers to improve the physical and mechanical properties of layered glued veneers during production, resulting in increased bending and tensile strength. Novosel et al. [19] examined the possibility of strengthening oak beams using CFRP sheets and epoxy resin, with hidden reinforcement through the use of wooden overlays. The load-bearing capacity and stiffness increased with the thickness and number of reinforcement layers, although the increase was not proportional. Sokołowski and Kossakowski [20] conducted research on reinforcing solid wooden beams with PBO fiber meshes, achieving a significant increase in load-bearing capacity with a relatively small increase in stiffness. Huang et al. [21] analyzed the influence of atmospheric and hydrothermal factors on the behavior of LVL beams with a hybrid glass-flax composite, observing a decrease in bending strength with an increase in beam ductility. Strzelecka et al. [22] conducted theoretical and numerical studies on the use of composite steel-veneer beams consisting of a steel section and an LVL plate. The behavior of layered wooden beams in fire conditions was presented in the work [23].

To sum up, the use of composite material as a passive reinforcement of wooden structures or as a face for wood-based core sandwich structures is a reasonable solution. It positively affects the static behavior of bent elements in terms of bending strength, stiffness, and ductility. The magnitude of that effect depends on many parameters such as the mechanical properties of wood and composites, the type of connection, and the position of the reinforcement.

This article introduces the concept of obtaining a new structural element in the form of a sandwich structure consisting of a core made of laminated veneer lumber, an epoxy resin layer used as a connector, and external surfaces with sheets of carbon fibers. A wood-based core is an environmentally friendly material that can easily be renewed and reused. The combination of epoxy resin and CFRP material is one of the most popular strengthening systems, and it is widely available on the market. The variables in the analysis were loading directions relative to the layer system and thicknesses of external surfaces. This study aims to verify the validity of using such a combination of materials in terms of the basic mechanical properties of the structure.

The presented novel sandwich structure is characterized by good mechanical properties and, therefore, can be competitive against traditional steel and reinforced concrete elements in civil engineering. In the described structure, common materials and a hand-laying technique were utilized. Therefore, it can be easily replicated without using special equipment. Depending on the orientation of veneers and CFRP in the structure, it can be utilized as beams, rafters, or slabs.

## 2. Materials and Methods

### 2.1. Materials

Experimental tests were performed using typical materials available on the Polish market. The CFRP sheet and epoxy resin are part of a strengthening system (offered by S&P Polsa Sp. z o.o. from Malbork, Poland) that was tested by the authors for strengthening timber structures in past experiments [14,15]; it is proven to be a reliable solution. The size of the tested specimens (LVL) was assumed taking into account the economic reasons and preliminary character of the study. A deeper analysis of the economical and esthetical aspects is required for full-size elements.

#### 2.1.1. Laminated Veneer Lumber

**Figure 1:**
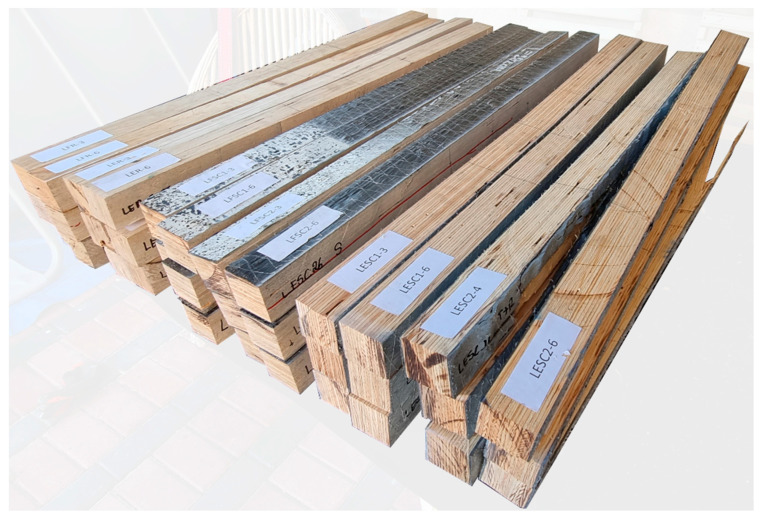
View of tested elements (photo made by the authors).

This study used laboratory-scale beams with nominal dimensions of 45 × 45 × 850 mm, consisting of 15 bonded veneers that were each approximately 3 mm thick. The tests were conducted in an edgewise (load applied to the plate’s edge) and flatwise configuration (load applied to the veneer surface). A view of the examined and, for the purposes of this work, unreinforced and reinforced samples is presented in Figure 1. Selected physical and mechanical properties of the laminated veneer lumber are shown in Table 1, providing a comparison of parameters obtained from the manufacturer’s data and the author’s own research. The physical and mechanical properties of LVL were evaluated according to [24,25]. After the tests, the moisture content of the beams was verified using an electro-porous moisture meter WRD-100 from Tanel Elektronika i Informatyka Spółka Jawna (Gliwice, Poland).

**Table 1:** Selected mechanical and physical parameters of laminated veneer lumber (prepared based on [14,15] according to the manufacturer’s data [26] and own experimental tests [27,28]).

Parameter	Value
Bending strength (edgewise condition) *f*_*m*,0,*edge*_ [MPa]	44 (80 ^1^)
Bending strength (flatwise condition) *f*_*m*,0,*flat*_ [MPa]	50 (78 ^1^)
Modulus of elasticity in bending (parallel to grain) *E*_0,*mean*_ [GPa]	14 (14.2 ^1^)
Tension strength (parallel to the grain) *f*_*t*,0,*k*_ [MPa]	36
Compression strength (parallel to the grain) *f*_*c*,0,*k*_ [MPa]	40 (58.5 ^1^)
Compression strength (perpendicular to grain) *f*_*c*,90,*k*_ [MPa]	7.5 (9.5 ^1^)
Mean value of density *ρ_d_* [kg/m^3^]	550 (600 ^1^)
Average moisture content of tested samples *w* [%]	13.3 ^1^

^1^ Based on own experimental tests.

In our own research, much higher values of mechanical parameters were obtained compared to the manufacturer’s data for the LVL plate. This is related to the scale of the tested elements in our research; tests were conducted on small samples. This phenomenon is consistent with the scale effect on the mechanics of structural wood failure [29]; as the volume of the tested element increases, its mechanical properties deteriorate. Only our own results were considered in further analyses.

#### 2.1.2. Carbon Fiber Reinforced Polymer (CFRP) Sheet

**Figure 2:**
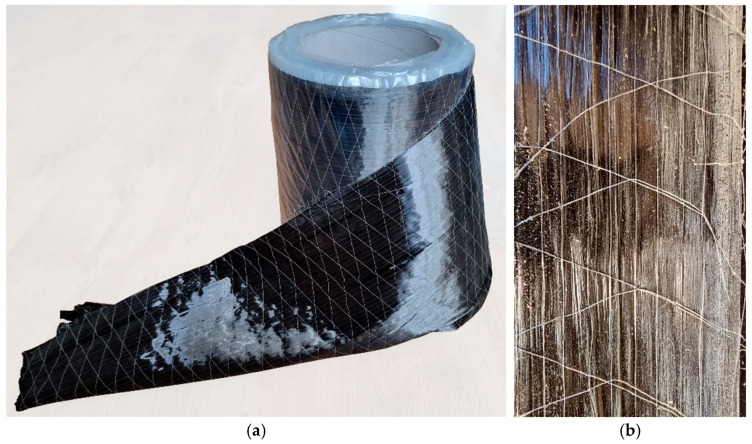
CFRP reinforcement: (**a**) CFRP sheet; (**b**) applied reinforcement (photo made by the authors).

As reinforcement, an unidirectionally reinforced CFRP carbon fiber sheet was used. The sheet was supplied in a roll with a width of 30 cm, which was then cut to the appropriate dimensions using scissors. The thickness of one layer of the sheet (reinforcement) was 0.333 mm, and for two layers, it was 0.666 mm. The sheets were placed on the gluing surface so that the direction of the carbon fiber was parallel to the direction of the wood grains in the veneers. Selected physical and mechanical properties of the CFRP sheet are presented in Table 2. The view of the sheet roll and the attached reinforcement is shown in Figure 2.

**Table 2:** Selected mechanical and physical parameters of CFRP (prepared based on [14,15,27,28] according to the manufacturer’s data [30]).

Parameter	Value
Modulus of elasticity *E_f_* [GPa]	265
Tensile strength *f*_*t*,*f*_ [MPa]	5100
Density *ρ_f_* [kg/m^3^]	1800
Elongation at rupture *ε_f_* [%]	1.7–1.9
Design thickness *t_f_* [mm]	0.333—one layer; 0.666—two layers

#### 2.1.3. Adhesive (Epoxy Resin)

To attach the composite material to the LVL core, a two-component, thixotropic epoxy resin-based adhesive was used, which was labeled S&P Resin 55 HP by the manufacturer [31]. The adhesive composition consisted of a solution of epoxy resin and amine hardener. The adhesive mixture was prepared by mixing the components in appropriate weight proportions using a slow-speed mixer. Then, the adhesive was applied to the cleaned surfaces of all joined elements. When two layers of CFRP sheet were used to increase the bonding strength, the quartz sand and epoxy layers were spread between them. The adhesive thickness on the veneer surface before applying the sheet was approximately 1 mm. After applying FRP material, the glue layer saturated it. The excess adhesive was removed with the use of a rubber spatula. In the end, no bonding layer can be distinguished on the cross-section using the naked eye. The adhesive consumption was about 1 kg per 1 m^2^ of joined surface. The binding reaction of epoxy resin is an exothermic reaction, and no external heating on joined components was applied during this process. Selected physical and mechanical properties of the adhesive are presented in Table 3—obtained according to [32,33,34].

**Figure 3:**
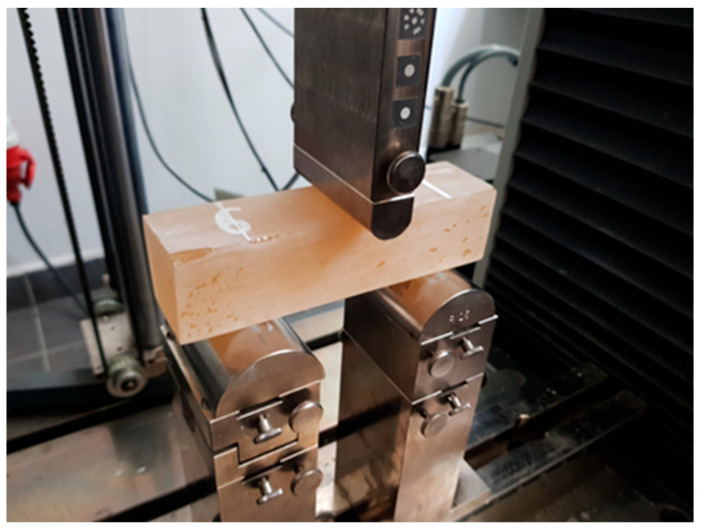
Bending strength test of epoxy resin (photo made by the authors).

Table 3 shows the results of the bending strength of the adhesive; the tests were conducted according to the standard [32] on 10 cuboidal samples with dimensions of 40 × 40 × 160 mm. The loading speed was 60 N/s. The view of the sample in the strength-testing machine is shown in Figure 3.

### 2.2. Methods

**Figure 4:**
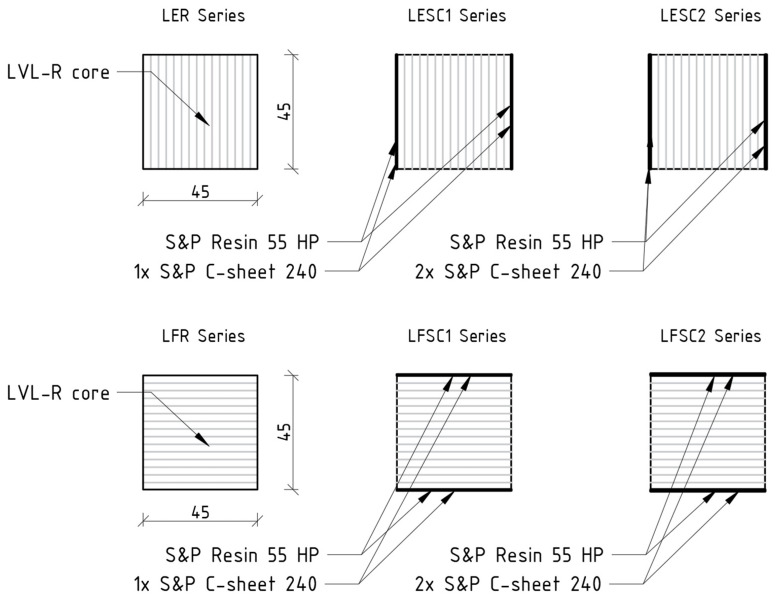
Strengthening configurations.

Laboratory tests were conducted at the Kielce University of Technology following the recommendations of the standards [24,25]. The aim of the research was to describe the static behavior of unreinforced and reinforced sandwich structure beams subjected to a 4-point bending test and introduce its possible application in civil engineering. The scope of the analysis involved preparation and bending till failure of the following test series (Figure 4):

- LER (Laminated Edgewise Reference)—unreinforced beams bent in an edgewise condition;
- LESC1 (Laminated Edgewise Strengthened Carbon-1-layer)—beams reinforced with one layer of CFRP sheet bonded to the sides of the core parallel to the load direction;
- LESC2 (Laminated Edgewise Strengthened Carbon-2-layers)—beams reinforced with two layers of CFRP sheet bonded to the sides of the core parallel to the load direction;
- LFR (Laminated Flatwise Reference)—unreinforced beams bent in a flatwise condition;
- LFSC1 (Laminated Flatwise Strengthened Carbon-1-layer)—beams reinforced with one layer of CFRP sheet bonded to the upper and lower surface of the core perpendicular to the load direction;
- LFSC2 (Laminated Flatwise Strengthened Carbon-2-layers)—beams reinforced with two layers of CFRP sheet bonded to the upper and lower surface of the core perpendicular to the load direction.

In total, 36 samples were tested, with 24 being reinforced elements and 12 reference elements.

**Figure 5:**
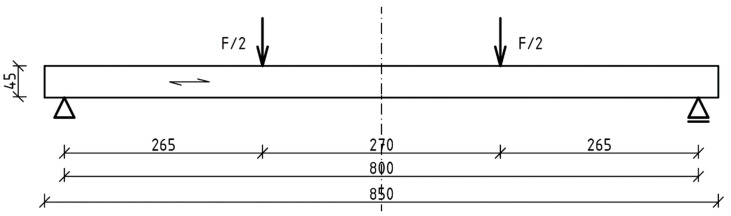
Scheme of the test stand.

**Figure 6:**
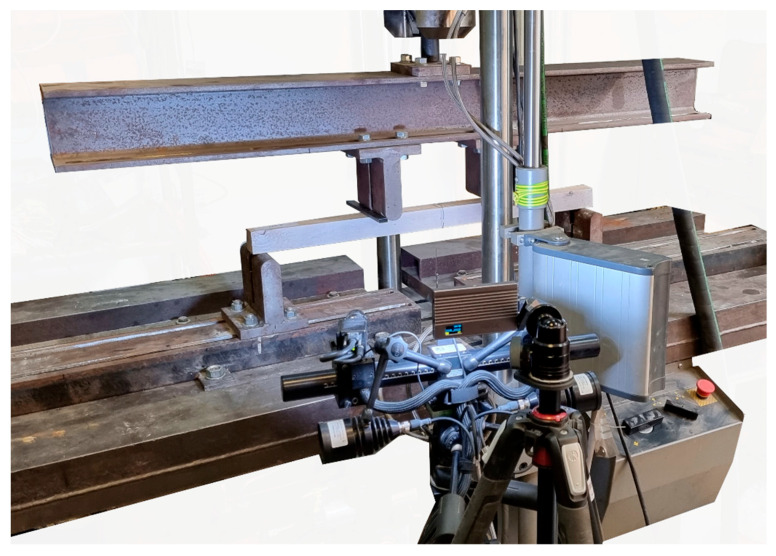
View of the test stand.

The schematic of the test stand is shown in Figure 5, and its view is shown in Figure 6. The beams were loaded symmetrically with two concentrated forces. The distance between the loading points was 270 mm. The distance from the support axis to the point of load application was 265 mm. The span of the beam in the support axes was 800 mm. The loading was controlled by the speed of the actuator displacement; the displacement speed was set at 3.5 mm/min. The actuator load was distributed over two concentrated forces using a steel I-beam. Steel guide plates with a width of 20 mm were applied at the point of concentrated force application. The steel guide plates were used to distribute the load over a larger surface area, preventing local damage due to veneer indentation.

During the tests, the loading force *F*, the test duration *t*, the actuator displacement using the MTS-322 universal testing machine with the load cell capacity equal to 100 kN (from MTS Systems Corporation, Eden Prairie, MN, USA), and the beam deflection in the middle of the span *u* using an inductive sensor were continuously recorded. After the tests, photographic documentation was prepared, and the destruction method was described.

## 3. Results and Analyses

The analysis of the static behavior of the beams was presented in relation to the bending strength, bending stiffness, and method of their destruction. Additionally, the possibility of applying a simple mathematical model and numerical analysis (FEM) to approximate the stiffness and bending strength of sandwich structure beams was verified.

### 3.1. Load-Bearing Capacity

**Figure 7:**
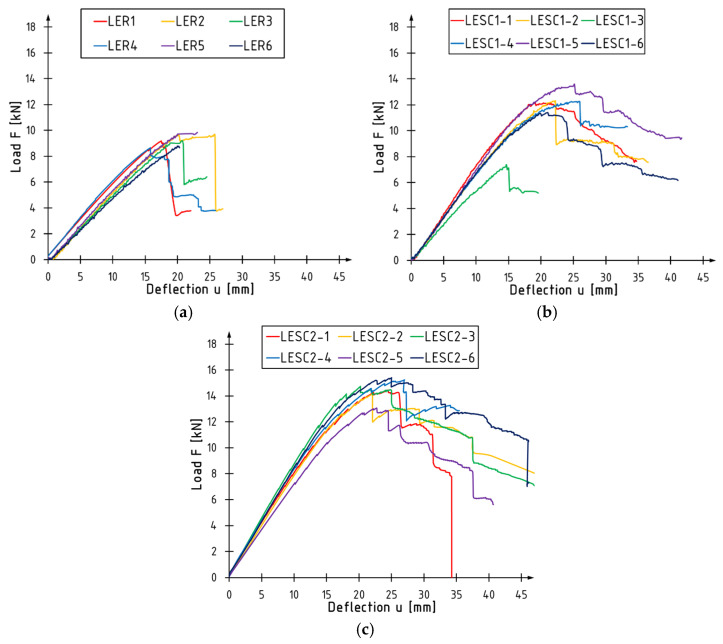
Load versus deflection curves for the beams tested in an edgewise condition: (**a**) LER series; (**b**) LESC1 series; (**c**) LESC2 series.

Load-deflection diagrams for beams tested in an edgewise configuration are presented in Figure 7, and detailed results are included in Table 4, Table 5 and Table 6. The tables present values of the maximum loading force, maximum bending moment, deflection at maximum loading force, and the failure mode, which is described later in the article. Unreinforced beams exhibit linear behavior from the beginning of the test until their destruction. For reinforced beams, plasticization of the cross-section occurs in the final phase of the test, caused by the propagation of destruction in the compressed zone of the core. Destruction of reinforced beams occurs abruptly, and with an increase in the degree of reinforcement, the plastic phase of work lengthens.

**Figure 8:**
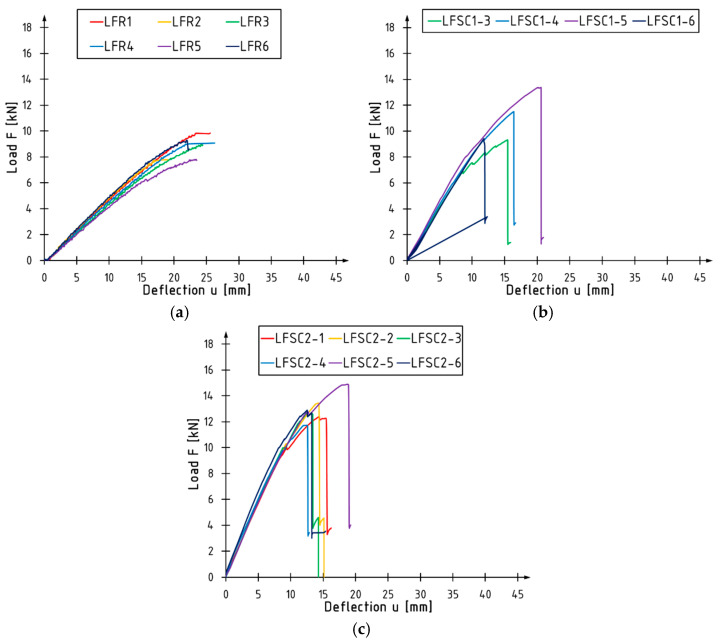
Load versus deflection curves for the beams tested in a flatwise condition: (**a**) LFR series; (**b**) LFSC1 series; (**c**) LFSC2 series.

Load-deflection diagrams for beams tested in a flatwise configuration are presented in Figure 8, and detailed results are included in Table 7, Table 8 and Table 9. The tables present similar data as for the previous configurations. Unreinforced beams exhibit a linear relationship between the increase in load and deflection. Reinforced elements behave similarly. In their case, in the final phase of the test, the curves undergo a slight incline from the initial course. However, attention should be paid to the significant increase in the stiffness of beams with additional layers of CFRP sheet. The destruction of reinforced beams occurs at a smaller deflection but with a higher value of the applied force compared to reference beams. Due to a measurement system failure, results for two elements from the LFSC1 series are not presented.

**Figure 9:**
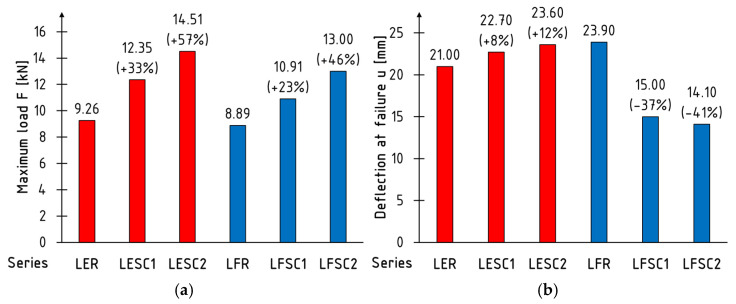
Average value: (**a**) maximum loading force F; (**b**) deflection at maximum loading force u.

Figure 9 summarizes the subsection in the form of histograms of the mean values of the maximum loading force and deflection at maximum loading force. Beams tested in edgewise and flatwise condition, are marked with red and blue color respectively. Results for the edgewise and flatwise configurations are marked in red and blue, respectively. The largest increases in bending strength were achieved with reinforcement placed on the sides of the core, amounting to 33% and 57% for one and two layers of reinforcement, respectively. Smaller increases in strength for reinforcement placed on the upper and lower surfaces, amounting to 23% and 46% for one and two layers of reinforcement, respectively, result from the destruction method of the system. Regardless of the analyzed CFRP-adhesive LVL configurations, destruction was always initiated in the wooden core. The damage to the veneer was a consequence of the destruction, leading to the impairment of the composite.

A significantly different behavior was observed in terms of deflection at maximum force. In the case of beams tested in a flat configuration, it underwent a substantial decrease, resulting from the increase in the system’s stiffness. In the edge configuration, reinforcement caused a slight increase in this parameter by approximately 10%.

The higher the reinforcement ratio is, the higher the increase of the load-bearing capacity is. However, the increase in the load-bearing capacity is not linear. Similar behavior was recorded in [15], where full-size LVL beams were strengthened with CFRP sheets. Considering the scale effect [14,15], a higher increase in the load-bearing capacity should be obtained for bigger sections.

### 3.2. Bending Stiffness

Bending stiffness coefficient k was evaluated according to the formula [35]:

k = F/u,(1)

where F is the loading force and u is the deflection at the midspan.

**Figure 10:**
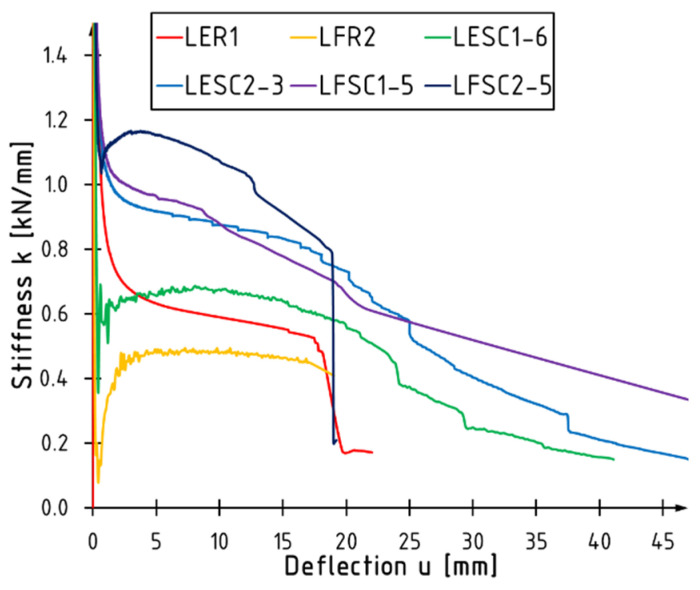
Bending stiffness versus deflection for selected tested beams.

Figure 10 presents curves showing changes in the stiffness of bent elements concerning the deflection increase in the middle of the span for selected elements from each tested series. For each beam, a decreasing stiffness was noted with the increasing applied force. The most significant changes occur when one of the composite components is damaged, and two types of changes can be distinguished: abrupt changes resulting from veneer cracking in the tensile zone or its shearing in the support zone (curves marked in red and navy blue) and gradual changes due to slow crack propagation in the compressed zone (curves marked in green and blue).

**Figure 11:**
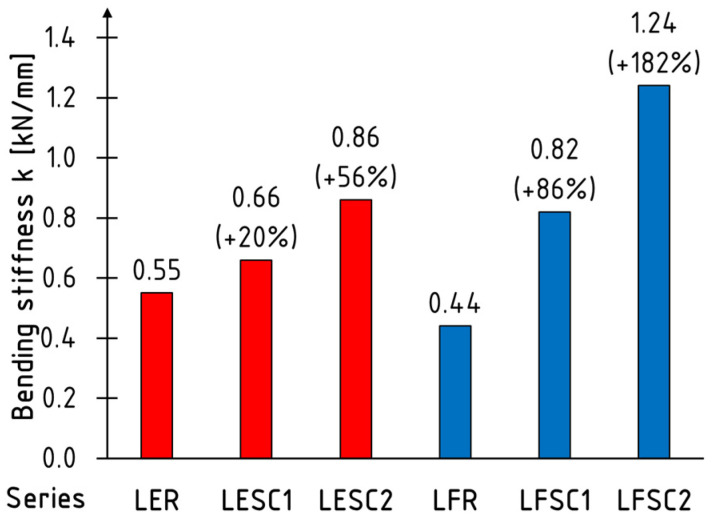
Average values of bending stiffness.

Due to the substantial variability in stiffness values throughout the study, it was decided to present its average value for each series calculated in the range from 0.1 to 0.4 of the maximum loading force; the results are plotted in Figure 11. Beams tested in edgewise condition are marked with red and in flatwise condition with blue color. The most significant stiffness increases were achieved when placing the reinforcement on the extremely compressed and tensile faces, amounting to 86% and 182% for the LFSC1 and LFSC2 series, respectively, compared to unreinforced beams. The location of the reinforcement on the side surfaces had a smaller impact on stiffness growth. However, increases in this parameter were also noted, which were approximately 20% and 56% for one and two layers of the sheet, respectively.

The bending stiffness of small-scale LVL specimens is smaller than the full-size one when compared with the results presented in [35,36]. This relationship is inverse to the load-bearing capacity—Table 1. The increase in bending stiffness is nonlinear with a linear increase in the reinforcement ratio.

### 3.3. Failure Modes

**Figure 12:**
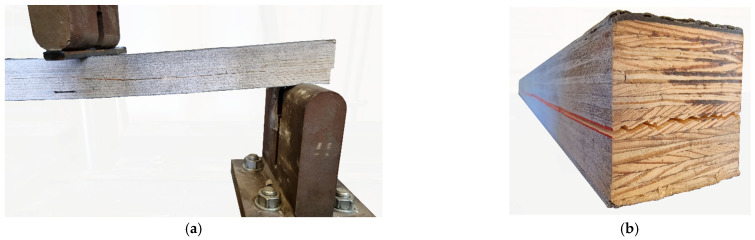

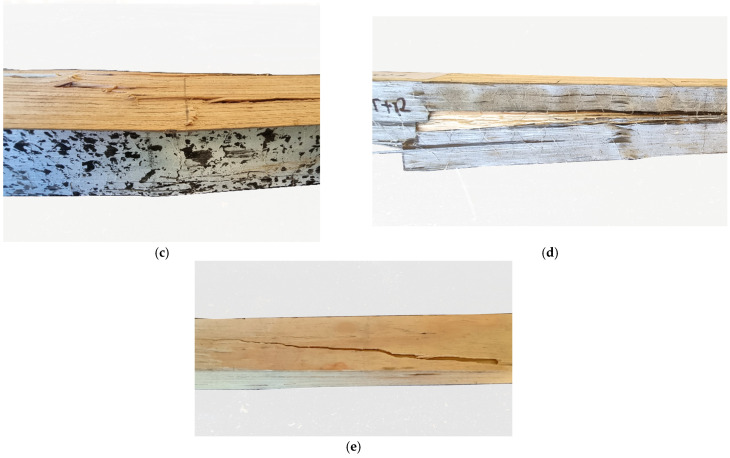
Failure modes: (**a**) shear failure—front view; (**b**) shear failure—axonometric view; (**c**) compression of veneers; (**d**) rupture of CFRI; (**e**) tensile failure of LVL.

Unreinforced beams, regardless of the testing direction, were destroyed due to exceeding the tensile strength in the tensile zone (typified as tension). This destruction was abrupt, unannounced, and usually resulted from a single crack encompassing many layers of veneers. The typical destruction of beams in an edge configuration was a slow degradation of the wooden core in the compressed zone (typified as compression), which was combined in the final phase of the test with the rupture of the carbon mat (rupture of composite) and wooden fibers in the tensile zone (tension). In this case, reinforcement improved work safety by signaling the critical state both visually through the wrinkling of external surfaces and acoustically in the form of cracking sounds. Reinforced beams in a flat configuration usually underwent destruction due to the shearing of core layers (typified as shear); similarly to unreinforced beams, this destruction was unannounced. Examples of destruction are shown in Figure 12.

Tension failure mode for unreinforced elements is a very common type that was reported in many publications when testing timber beams [14,15,27,35,36]. However, the destruction of FRP is not a typical failure for a reinforced section [37,38]. It occurs when the reinforcement ratio is relatively low or when the reinforcement layer is thin [39]. More common, but not observed in the presented paper, is the failure of the adhesive layer causing the debonding of FRP material.

### 3.4. Numerical Analysis

The numerical model of unreinforced and reinforced laminated veneer beams was prepared in the standard module of Abaqus 2017 software. In this subsection, only the most important assumptions adopted for its construction are included, and their results are synthetically described. The methodology used was based on guidelines and experiences drawn from numerical analyses described by other scientists, as presented in [40,41].

**Figure 13:**
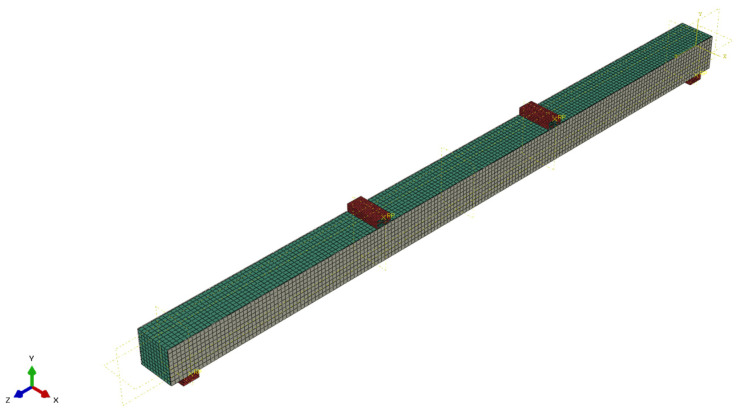
View of the modeled beam in Abaqus software.

The core was modeled as a three-dimensional solid element, supports were modeled as discrete rigid elements, and external surfaces were modeled as three-dimensional shell elements. An elastic-perfectly plastic material model was adopted for LVL and a linear model for CFRP sheet. Material model parameters were adopted according to Table 1 and Table 2. The connection between external surfaces and the core was made using tie constraints, neglecting the influence of the adhesive layer on static behavior. Loading was controlled by the displacement of the loading pressure. The calculations were performed in the static range. An approximate global finite element size of 5 mm was adopted. The following types of finite elements were used: core—C3D8R; CFRP sheet—S4R; and supports—R3D4. A view of the assembled FEM model is shown in Figure 13.

**Figure 14:**
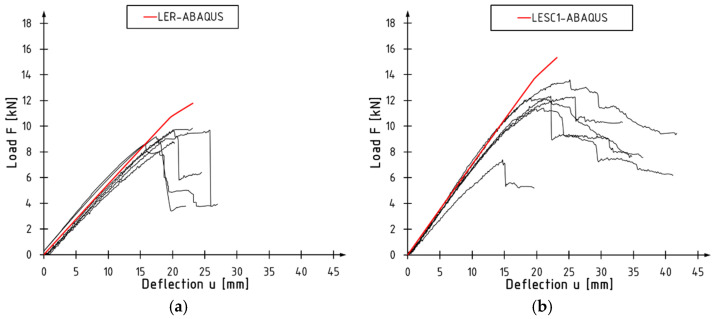
Load versus deflection curves: (**a**) LER series; (**b**) LESC1 series.

Figure 14 presents curves obtained from Abaqus 2017 against the results of experimental tests for a selected unreinforced and reinforced series. The adopted model accurately describes the behavior of the tested elements in the linear range, with differences not exceeding 10%. Larger discrepancies occur in the final phase of the test, where the plasticization of the plastic section occurs.

### 3.5. Mathematical Model

To determine the theoretical values of stiffness and the maximum bending moment, the method of equivalent cross-section was used. In this method, the material on the external surfaces was accounted for by proportionally increasing the dimensions of the core cross-section. This increase depends on the ratio of the modulus of elasticity of the reinforcement to the modulus of elasticity of the reinforced material. The assumptions and principles of creating this model were described in detail in the papers [42,43,44,45,46]. Here, only its most important assumptions and analysis results are presented.

The proportionality coefficient n was evaluated according to the following formula:

(2) $$n=\frac{E_{FRP}}{E_{LVL}},$$

where E_FRP_ is the modulus of elasticity of FRP and E_LVL_ is the modulus of elasticity of LVL.

The position of the neutral axis z_c_ was evaluated according to the following formula:

(3) $$z_{c}=\frac{\sum_{i=1}^{n}A_{i}\cdot z_{i}}{\sum_{i=1}^{n}A_{i}}$$

where A_i_ is the cross-section area of each component figure and z_i_ is the distance from the center of component figure to the position of the y-axis.

The second moment of inertia of the transformed cross-section at the y-axis was evaluated according to the following formula:

(4) $$I_{y}=\sum_{i=1}^{n}\left(I_{yi}+A_{i}\cdot {\left(z_{i}-z_{c}\right)}^{2}\right)$$

where I_yi_ is the second moment of inertia of each component figure.

The maximum bending moment for transformed cross section M was evaluated according to the following formula:

(5) $$f_{m}=\frac{M\cdot z}{I_{y}}\to M=\frac{f_{m}\cdot I_{y}}{z}$$

where f_m_ is the bending strength of LVL and z is the distance between the neutral axis and the extreme compressed face.

The bending stiffness EI_theo_ was evaluated according to the following formula:

(6) $$EI_{theo}=I_{y}\cdot E_{LVL}.$$

**Figure 15:**
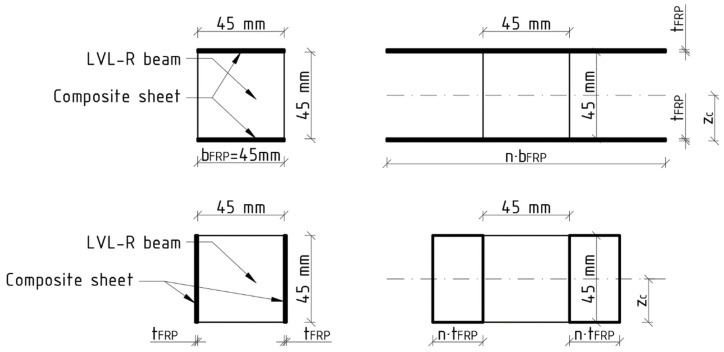
View of transformed cross-sections.

The transformed cross-section for the edgewise and flatwise conditions are shown in Figure 15.

**Figure 16:**
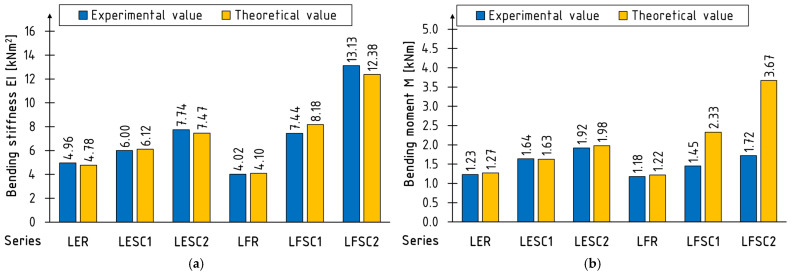
Comparison of the theoretical and experimental values of the (**a**) bending stiffness EI and (**b**) the bending moment M.

The analytical analysis results are shown in Figure 16. A very good agreement was achieved between experimental and theoretical values of bending stiffness for all tested series. In the case of bending strength, the model proved useful for estimating bending strength only in the edge configuration. Due to the premature destruction of the structure resulting from veneer shearing, it should not be applied to the flat configuration; the obtained theoretical values are even more than twice as high as the experimental ones. Another approach to consider is protecting the flat configuration from this form of destruction and only then testing the feasibility of its application.

A high agreement of the estimated bending stiffness and the bending moment of strengthened timber beams with experimental results using a transformed cross-section despite its simplicity is very common [42,47]. Soriano et al. [48] suggested that this method may be inadequate for reinforcement ratios higher than 4%, for timber beams strengthened with steel bars. More complex theoretical models involve incorporating different distributions of stress along the depth of cross-sections [49,50]. A strain-based procedure for designing strengthened timber beams with FRP is presented in [51].

## 4. Conclusions

This article presents preliminary results of experimental research on the static behavior of bent specimens with a sandwich structure, with a core made of laminated veneer and external surfaces made of CFRP sheet. The tests were carried out on laboratory-scale beams. The following was found:

1. The presented novel sandwich structure is characterized by significantly better mechanical properties than the core alone and can be competitive against traditional elements. Further tests, however, are required in order to evaluate the scale effect with different core sizes. From an economical point of view, we propose incorporating cheaper FRP materials like aramid, basalt, and glass sheet and evaluating their influence on the mechanical properties of sandwich structures.
2. The greatest load-bearing capacity was achieved when the reinforcement was placed on the sides of the core, parallel to the veneer layers in the core, and in the direction of the applied load, which led to the highest utilization of core strength. The greatest bending stiffness was achieved by placing reinforcement on the extremely compressed and tensile surfaces, i.e., when the center of gravity of the reinforcement was as far away as possible from the center of gravity of the core.
3. In experimental tests, only external reinforcement was analyzed. For such configuration, the influence of elevated temperatures and post-fire mechanical properties as well as long-term behavior in various environmental conditions should be considered in future tests.
4. The failure of the core alone results from cracking in the tensile zone. The failure of the sandwich structure results from shearing or compression of the core, or rupture of the external layer. Investigation of the possible ways of preventing the most common shear failure mode occurring in flatwise conditions is needed.
5. The weakest element in the CFRP-LVL sandwich structure is the core made of laminated veneer; therefore, its arrangement concerning the direction of the applied force should be considered during the design stage of structural elements. No premature failure, due to the failure of connection, was recorded, and a high-quality bond was obtained with the use of epoxy resin.
6. The possibility of using a numerical analysis and simple mathematical model to evaluate beam stiffness has been confirmed. However, the applicability of a mathematical model for estimating bending strength is limited to situations where destruction is not caused by shearing.

## Figures and Tables

**Table 3:** Selected mechanical and physical parameters of epoxy resin S&P Resin 55 HP (prepared based on [14,15,27] according to the manufacturer’s data [31] and own experimental tests [28]).

Parameter	Value
Bending strength *f_m_* [MPa]	85.3 ^1^
Modulus of elasticity *E_k_* [MPa]	3200
Density *ρ_k_* [kg/m^3^]	1200–1300 (1150 ^1^)
Compressive strength *f*_*c*,*k*_ [MPa]	100

^1^ Based on own experimental tests.

**Table 4:** LER series test results.

Parameter	Beam						Mean Value
	LER1	LER2	LER3	LER4	LER5	LER6	
*F_max_* [kN]	9.19	9.68	9.20	8.67	9.86	8.94	9.26
*M_max_* [kN]	1.22	1.28	1.22	1.15	1.31	1.19	1.23
*u_max_* [mm]	17.5	25.8	20.8	15.8	23.0	23.2	21.0
Failure mode	Tension	Tension	Tension	Tension	Tension	Tension	-

**Table 5:** LESC1 series test results.

Parameter	Beam						Mean Value
	LESC1-1	LESC1-2	LESC1-3 ^1^	LESC1-4	LESC1-5	LESC1-6	
*F_max_* [kN]	12.17	12.33	7.38	12.27	13.59	11.40	12.35
*M_max_* [kN]	1.61	1.63	0.98	1.63	1.80	1.51	1.64
*u_max_* [mm]	19.1	22.2	14.7	25.8	25.2	21.0	22.7
Failure mode	Tension + Compression + Rupture of composite	Tension + Compression + Rupture of composite	Tension + Rupture of composite	Tension + Rupture of composite	Tension + Compression + Rupture of composite	Tension + Compression + Rupture of composite	-

^1^ Omitted in statistical analysis due to a big difference in comparison to other elements in the series.

**Table 6:** LESC2 series test results.

Parameter	Beam						Mean Value
	LESC2-1	LESC2-2	LESC2-3	LESC2-4	LESC2-5	LESC2-6	
*F_max_* [kN]	14.45	14.17	14.75	15.24	13.06	15.41	14.51
*M_max_* [kN]	1.92	1.88	1.95	2.02	1.73	2.04	1.92
*u_max_* [mm]	24.5	22.1	20.2	27.0	22.7	25.0	23.6
Failure mode	Tension + Compression + Rupture of composite	Tension + Compression + Rupture of composite	Tension + Compression + Rupture of composite	Tension + Rupture of composite	Tension + Compression + Rupture of composite	Tension + Rupture of composite	-

**Table 7:** LFR series test results.

Parameter	Beam						Mean Value
	LFR1	LFR2	LFR3	LFR4	LFR5	LFR6	
*F_max_* [kN]	9.92	7.97	9.05	9.12	7.96	9.32	8.89
*M_max_* [kN]	1.32	1.06	1.20	1.21	1.05	1.23	1.18
*u_max_* [mm]	25.6	19.5	26.4	26.3	23.5	21.9	23.9
Failure mode	Tension	Tension	Shear	Tension	Tension	Tension	-

**Table 8:** LFSC1 series test results.

Parameter	Beam						Mean Value
	LFSC1-1 ^1^	LFSC1-2 ^1^	LFSC1-3	LFSC1-4	LFSC1-5	LFSC1-6	
*F_max_* [kN]	-	-	9.32	11.52	13.37	9.43	10.91
*M_max_* [kN]	-	-	1.24	1.53	1.77	1.25	1.45
*u_max_* [mm]	-	-	17.5	11.3	19.6	11.8	15.0
Failure mode	Shear	Shear	Shear + Rupture of composite	Shear	Shear	Shear	-

^1^ Unavailable result due to the failure of the measuring system.

**Table 9:** LFSC2 series test results.

Parameter	Beam						Mean Value
	LFSC2-1	LFSC2-2	LFSC2-3	LFSC2-4	LFSC2-5	LFSC2-6	
*F_max_* [kN]	12.37	13.40	12.75	11.71	14.85	12.90	13.00
*M_max_* [kN]	1.64	1.78	1.69	1.55	1.97	1.71	1.72
*u_max_* [mm]	14.3	14.3	12.5	12.3	18.4	12.5	14.1
Failure mode	Shear	Shear	Shear	Shear	Shear	Shear	-

## Data Availability

Data are contained within the article.
